# Urine cell-free DNA multi-omics to detect MRD and predict survival in bladder cancer patients

**DOI:** 10.1038/s41698-022-00345-w

**Published:** 2023-01-19

**Authors:** Pradeep S. Chauhan, Alexander Shiang, Irfan Alahi, R. Taylor Sundby, Wenjia Feng, Bilge Gungoren, Cayce Nawaf, Kevin Chen, Ramandeep K. Babbra, Peter K. Harris, Faridi Qaium, Casey Hatscher, Anna Antiporda, Lindsey Brunt, Lindsey R. Mayer, Jack F. Shern, Brian C. Baumann, Eric H. Kim, Melissa A. Reimers, Zachary L. Smith, Aadel A. Chaudhuri

**Affiliations:** 1 Division of Cancer Biology, Department of Radiation Oncology, Washington University School of Medicine, St. Louis, MO USA; 2 Division of Urology, Department of Surgery, Washington University School of Medicine, St. Louis, MO USA; 3 Department of Computer Science and Engineering, Washington University in St. Louis, St. Louis, MO USA; 4 Pediatric Oncology Branch, Center for Cancer Research, National Cancer Institute, National Institutes of Health, Bethesda, MD USA; 5 Division of Chemistry and Chemical Engineering, California Institute of Technology, Pasadena, CA USA; 6 Siteman Cancer Center, Barnes Jewish Hospital and Washington University School of Medicine, St. Louis, MO USA; 7 Wilmot Institute Cancer Center, University of Rochester Medical Center, Rochester, NY USA; 8 Division of Medical Oncology, Department of Medicine, Washington University School of Medicine, St. Louis, MO USA; 9 Department of Biomedical Engineering, Washington University in St. Louis, St. Louis, MO USA; 10 Department of Genetics, Washington University School of Medicine, St. Louis, MO USA

**Keywords:** Bladder cancer, Tumour biomarkers, Next-generation sequencing, Next-generation sequencing, Cancer genomics

## Abstract

Circulating tumor DNA (ctDNA) sensitivity remains subpar for molecular residual disease (MRD) detection in bladder cancer patients. To remedy this problem, we focused on the biofluid most proximal to the disease, urine, and analyzed urine tumor DNA in 74 localized bladder cancer patients. We integrated ultra-low-pass whole genome sequencing (ULP-WGS) with urine cancer personalized profiling by deep sequencing (uCAPP-Seq) to achieve sensitive MRD detection and predict overall survival. Variant allele frequency, inferred tumor mutational burden, and copy number-derived tumor fraction levels in urine cell-free DNA (cfDNA) significantly predicted pathologic complete response status, far better than plasma ctDNA was able to. A random forest model incorporating these urine cfDNA-derived factors with leave-one-out cross-validation was 87% sensitive for predicting residual disease in reference to gold-standard surgical pathology. Both progression-free survival (HR = 3.00, *p* = 0.01) and overall survival (HR = 4.81, *p* = 0.009) were dramatically worse by Kaplan–Meier analysis for patients predicted by the model to have MRD, which was corroborated by Cox regression analysis. Additional survival analyses performed on muscle-invasive, neoadjuvant chemotherapy, and held-out validation subgroups corroborated these findings. In summary, we profiled urine samples from 74 patients with localized bladder cancer and used urine cfDNA multi-omics to detect MRD sensitively and predict survival accurately.

## Introduction

**Fig. 1:**
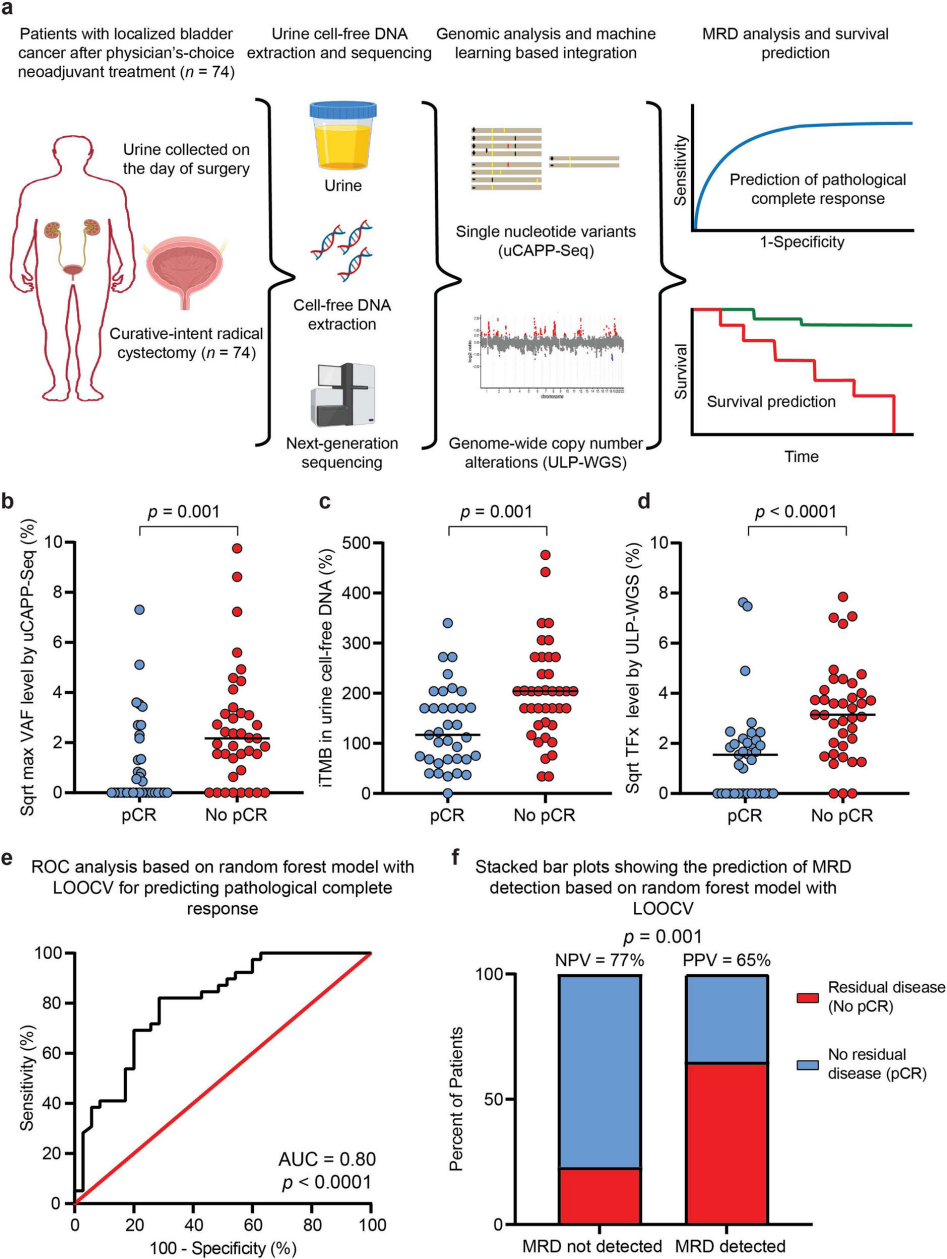
Pathologic complete response prediction using a random forest model based on urine tumor DNA. **a** Urine was collected prospectively from 74 localized bladder cancer patients pre-operatively on the day of curative-intent radical cystectomy after physician’s-choice neoadjuvant treatment. Urine cell-free DNA was sequenced by uCAPP-Seq (for single nucleotide variants) and ULP-WGS (for genome-wide copy number alterations) and then correlated with residual tumor in the surgical resection specimen and with patient survival. This figure panel was created with BioRender.com. **b** SNV-derived maximum VAFs, **c** inferred tumor mutational burden, and **d** CNA-derived tumor fraction levels in urine cell-free DNA from patients with localized bladder cancer. Scatter plots display these three different urine cell-free DNA metrics, stratified by pathologic complete response status, with significance determined by the Mann–Whitney *U*-test. VAF and CNA-derived tumor fraction data are shown after square root transformation. **e** ROC analysis of random forest model integrating urine tumor DNA metrics and other pretreatment clinical variables (Supplementary Fig. 5). ROC curve demonstrating the model’s performance for predicting pCR after LOOCV (AUC = 0.80, *p* < 0.0001). **f** Stacked bar plot depicting NPV and PPV of the random forest model with LOOCV, with significance determined by the Fisher’s exact test. AUC area under the curve, cfDNA cell-free DNA, CNA copy number alteration, iTMB inferred tumor mutational burden, LOOCV leave-one-out cross-validation, max maximum, MRD molecular residual disease, NPV negative predictive value, pCR pathologic complete response, PPV positive predictive value, ROC receiver operating characteristic, SNV single nucleotide variant, Sqrt square root, TFx tumor fraction, uCAPP-Seq urine cancer personalized profiling by deep sequencing, ULP-WGS ultra-low-pass whole genome sequencing, VAF variant allele frequency.

Bladder cancers shed tumor DNA into the urine, which can be measured using ultra-deep targeted sequencing^1,2,3^. However, the modest sensitivity of this approach to detect molecular residual disease (MRD) limits clinical utility^3^. Here, we analyzed urine cell-free DNA (cfDNA) using combinatorial ultra-deep targeted sequencing and ultra-low-pass whole genome sequencing (ULP-WGS) to sensitively detect MRD in urine and predict survival after curative-intent radical cystectomy (Fig. 1a and Supplementary Fig. 1).

## Results

### Cohort characteristics and biofluid samples

Seventy-four localized bladder cancer patients underwent a physician’s-choice of neoadjuvant treatment and curative-intent radical cystectomy. Seventy-eight percent (58/74) harbored muscle-invasive bladder cancer, while the rest had treatment-refractory non-muscle-invasive bladder cancer (Supplementary Data 1). Ninety-two percent (68/74) had urothelial carcinoma, while the remainder had variant histologies. A full description of the cohort is displayed in Supplementary Data 2. Urine cancer personalized profiling by deep sequencing (uCAPP-Seq) libraries prepared from urine cfDNA samples were sequenced to >900x median unique depth (Supplementary Data 3) along with comparably sequenced plasma (Supplementary Data 4) and germline DNA (Supplementary Data 5). ULP-WGS libraries prepared from urine cfDNA were sequenced to a median unique coverage of 2x (Supplementary Data 6).

### Cell-free DNA biomarker differences in relation to pCR status

Copy number-derived tumor fraction (TFx) levels, estimated from ULP-WGS of urine cfDNA, ranged from 0 to 62% with a median value of 4.3% in this cohort (Supplementary Data 2). Genome-wide analysis of urine cfDNA revealed focal copy number alteration of genes previously reported by The Cancer Genome Atlas (TCGA) to be recurrently altered in MIBC (Supplementary Fig. 2)^4,5^, with *PPARG, ZNF703*, and *E2F3* being the most frequently amplified. Further, uCAPP-Seq analysis of single nucleotide variant (SNV) data from our full 74 patient cohort revealed that the *TERT* promotor and *TP53* were the most commonly mutated genes (Supplementary Fig. 3), again consistent with prior tissue sequencing data^4–6^. Indicative of specificity, neither copy number alterations nor SNVs were detected with significance in healthy adult urine cfDNA (Supplementary Figs. 2, 3). Additionally, results of our copy number (Supplementary Fig. 2) and uCAPP-Seq (Supplementary Fig. 3) analyses demonstrated clear differences in urine cfDNA based on pathologic complete response (pCR) status, which was determined by examination of surgical specimens by board-certified genitourinary pathologists.

Bladder cancer patients who achieved pCR had significantly lower variant allele frequency (VAF) levels measured by uCAPP-Seq compared to those who did not (Fig. 1b) despite having similar baseline characteristics (Supplementary Data 7). Strikingly, urine cfDNA significantly outperformed plasma circulating tumor DNA (Supplementary Fig. 4). We also measured the tumor mutational burden inferred from the number of non-silent mutations detected in urine cfDNA (iTMB). The median iTMB was 170 (range 0–476) across the cohort, consistent with previous reports in bladder cancer^7^. Comparing between subgroups, patients with no pCR had significantly higher iTMB levels than patients with pCR (median 204 vs. 117, *p* = 0.001) (Fig. 1c). This result is consistent with findings in breast cancer, suggesting that increased TMB is a negative predictor of pCR to neoadjuvant chemotherapy^8^. TFx, which was inferred from genome-wide copy number alterations in urine cfDNA, also differed significantly based on pCR status (median 2.4% for pCR vs. 9.9% for no pCR, *p* < 0.0001) (Fig. 1d), suggesting that genome-wide copy number alterations, like SNVs, could be utilized for urine-based MRD detection in bladder cancer.

### Random forest model for pCR and survival prediction

We next integrated the three urine cfDNA-derived metrics—maximum VAF, iTMB, and TFx—with pretreatment clinical variables using a machine learning random forest model that we validated by leave-one-out cross-validation (LOOCV) (Supplementary Fig. 5a). Area under the receiver operating characteristic curve (AUROC) for the random forest model was 0.80 (*p* < 0.0001) (Fig. 1e), with a sensitivity of 87%, a negative predictive value (NPV) of 77%, and a positive predictive value (PPV) of 65% for determining pCR (Fig. 1f). The combinatorial urine cfDNA metric was by far the most important predictive feature in the model (Supplementary Fig. 5b). Indeed, when we developed a LOOCV model including only urine cfDNA features (maximum VAF, iTMB, and TFx), its performance remained high with AUROC of 0.76 for determining pCR (Supplementary Fig. 6).

**Fig. 2:**
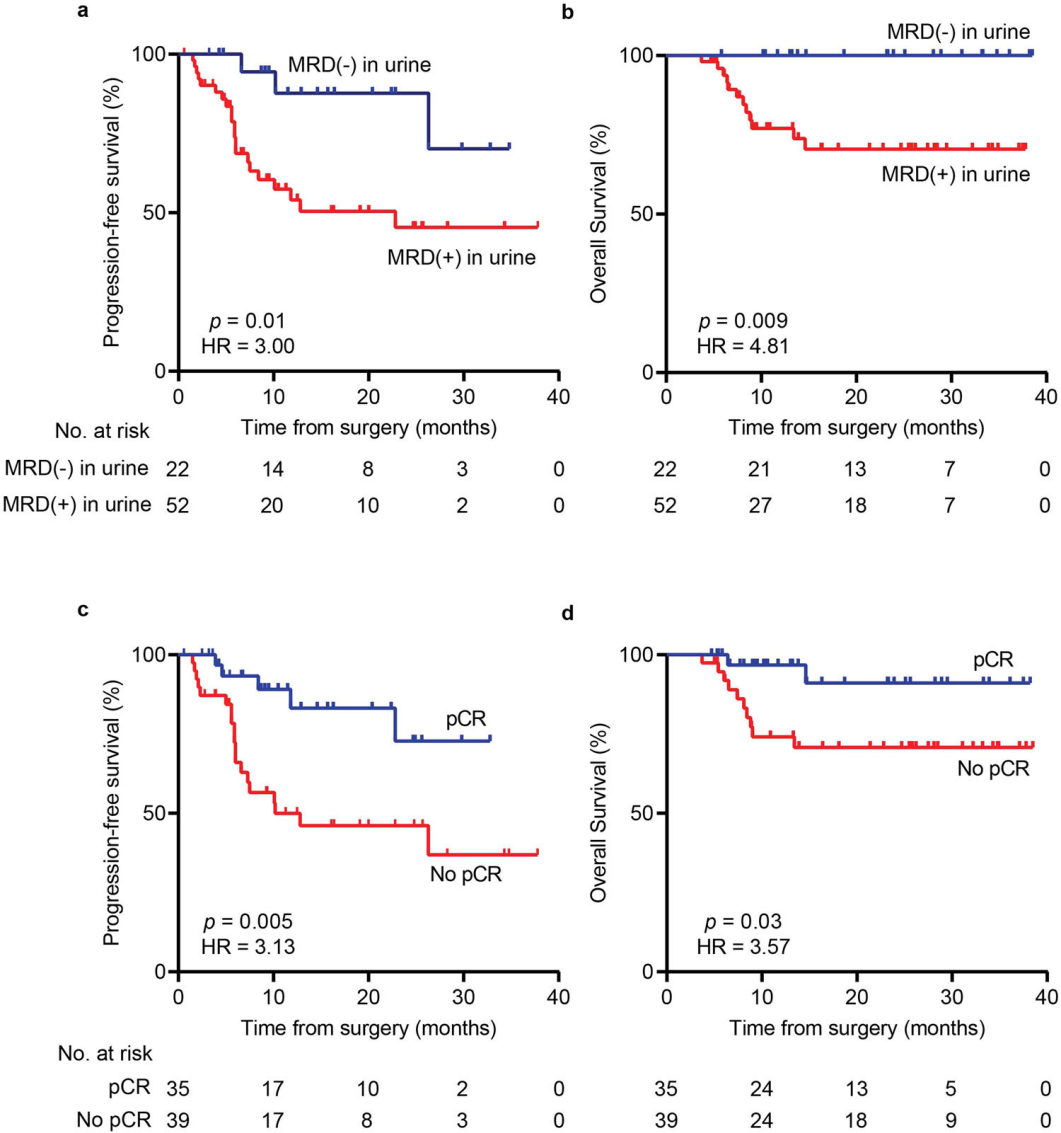
Survival analysis comparing urine MRD detection to pathologic analysis of the resection specimen. Kaplan–Meier plots showing **a** progression-free survival and **b** overall survival stratified by MRD detection in urine, determined by the LOOCV random forest model (Supplementary Fig. 5). **c** Progression-free survival and **d** overall survival stratified by pCR determined by microscopic analysis of the radical cystectomy specimen. Survival times shown are relative to the time of radical cystectomy. *p* values were calculated by the log-rank test and HRs by the Mantel–Haenszel method. HR hazard ratio, LOOCV leave-one-out cross-validation, MRD molecular residual disease, pCR pathologic complete response.

Using our LOOCV model, we also aimed to predict survival outcomes within our 74-patient localized bladder cancer cohort. Therefore, we performed Kaplan–Meier and Cox regression landmark analyses starting from the time of surgery (Fig. 2 and Supplementary Data 8, 9). Strikingly, patients predicted by our model to harbor MRD also had significantly worse progression-free survival (PFS) (HR = 3.00, *p* = 0.01; Fig. 2a) and overall survival (OS) (HR = 4.81, *p* = 0.009; Fig. 2b), comparable to the presence of residual disease in the radical cystectomy specimen itself (PFS HR = 3.13, *p* = 0.005; OS HR = 3.57, *p* = 0.03; Fig. 2c, d). Univariate and multivariate Cox proportional hazards models confirmed the significance of our MRD predictions (Supplementary Data 8, 9). The model remained predictive for both PFS and OS when restricted to only MIBC patients (Supplementary Fig. 7) and patients treated with NAC (Supplementary Fig. 8). Furthermore, the model remained significant for predicting PFS when applied to an independent held-out validation cohort (Supplementary Fig. 9a) with a trend toward predicting OS significantly as well (Supplementary Fig. 9b).

## Discussion

Here, we developed a multi-modal urine cfDNA method to sensitively detect MRD and predict pCR in bladder cancer patients. Our technology also predicted survival significantly and comparably to gold-standard surgical pathologic analysis of resected tumor tissue^9^. Limitations of our study include patients having only a single timepoint assessment of urine cfDNA. Other investigations utilizing plasma have shown that multiple samples obtained in surveillance settings can achieve greater sensitivity for detecting circulating tumor DNA MRD^10,11^. We nevertheless achieved high MRD sensitivity by multimodally analyzing urine, the biofluid most proximal to localized bladder cancer. While our study was prospective, all samples were obtained from a single medical center. It will be important to corroborate our findings in a multi-institutional setting. Finally, given the prospective nature of our study with all patients enrolled between 2019 and 2021, the median follow-up time was modest at 23 months. It will be important to perform a study with a longer follow-up to confirm the dramatic survival differences we observed.

In conclusion, our multi-omic urine-based cell-free DNA analysis allowed for the detection of MRD with high sensitivity and risk-stratified patients by survival. In the future, this type of integrative analysis could potentially be used to facilitate more personalized clinical decision-making for bladder cancer.

## Methods

### Patient recruitment and sample collection

We enrolled 74 patients with localized bladder cancer who proceeded with curative-intent radical cystectomy at the Washington University Siteman Cancer Center. Eligible patients were required to be at least 18 years old and to have a diagnosis of bladder cancer confirmed by histologic or cytologic assessment. Urine and blood collection was performed at the time of enrollment. We also utilized urine and blood samples from 15 healthy adult volunteers for comparison. The methods were performed in accordance with relevant guidelines and regulations and approved by the institutional review board at the Washington University in St. Louis School of Medicine. Patients and healthy donors were enrolled in NCT04354064 (ClinicalTrials.gov). Written informed consent was obtained from all trial participants in accordance with the Declaration of Helsinki. This study followed the Strengthening the Reporting of Observational Studies in Epidemiology (STROBE) guidelines for observational studies.

### Pathologic response assessment

Surgical resection specimens from radical cystectomy procedures were processed consistently using a standardized institutional approach, including specimen collection, handling, and submission to the Pathology Department at the Washington University School of Medicine. Resected surgical specimens were microscopically reviewed by blinded board-certified genitourinary surgical pathologists. AJCC 8th edition pathologic stage T0, Tis, and Ta were defined as pathologic complete response (pCR) in our study. Non-pathologic complete response (no pCR) was defined as stages T1, T2, T3, or T4, with or without evidence of nodal disease (N1–N2) and/or evidence of metastatic disease.

### Urine cell-free DNA extraction

Urine samples were collected in cups pre-filled with 1–2 mL of 0.5 M EDTA. Shortly following collection, cfDNA was extracted from 22 to 90 ml of urine with Q-sepharose resin slurry (GE Healthcare, Chicago, Illinois)^3^. Briefly, Q-sepharose resin was added to urine at a ratio of 10 ul slurry per ml of urine and mixed for 30 min. After centrifuging the mixture at 1800 × *g* for 10 min, the supernatant was discarded. The resin was washed twice with 0.3 M LiCl/10 mM sodium acetate (pH 5.5), transferred to a Micro Bio-Spin column (Bio-Rad, Hercules, California, USA), and the bound DNA was eluted with 70% ethanol and passed over a QIAquick column (Qiagen, Hilden, Germany). Columns were then washed with 2 M LiCl in 70% ethanol, followed by 75 mM potassium acetate (pH 5.5) in 80% ethanol. Finally, DNA was eluted in nuclease-free water or 10 mM Tris-Cl (pH 8.5). Urine cfDNA was quantified using the Qubit dsDNA High Sensitivity Assay kit (Thermo Fisher Scientific, Waltham, Massachusetts). cfDNA quality was assessed on an Agilent 2100 Bioanalyzer (Agilent Technologies, Santa Clara, California).

### Germline DNA extraction

A peripheral blood sample was collected from each subject using EDTA tubes (Becton Dickinson, Franklin Lakes, New Jersey). Plasma-depleted whole blood (PDWB) was collected by centrifugation and then frozen at −80 °C prior to the isolation of germline DNA. Germline DNA was extracted from 50 to 100 ul of PDWB using the QIAmp DNA Micro Kit (Qiagen, Hilden, Germany) according to the manufacturer’s instructions. DNA was then quantified by the Qubit dsDNA High Sensitivity Assay to determine yield (Thermo Fischer, Waltham, Massachusetts).

### Cancer personalized profiling by deep sequencing (CAPP-Seq)

Urine CAPP-Seq was performed on urine cfDNA along with matched germline DNA^1,3^. Briefly, urine cfDNA and germline DNA were fragmented to ~180 bp size fragments prior to library preparation using a LE220-focused ultrasonicator (Covaris, Woburn, Massachusetts). Approximately 32 ng of sheared urine cfDNA or germline DNA was used for library preparation using the KAPA HyperPrep kit with barcoded adapters containing demultiplexing, deduplicating, and duplexed unique molecular identifiers. Targeted hybrid capture was performed per the standard uCAPP-Seq method^1,3^. We used a focused MRD gene panel spanning 145 kb in size and consisting of 49 consensus driver genes frequently mutated in bladder cancer for the VAF estimation in each sample^3^. For TMB estimation, we utilized an expanded panel of 387 kb in size which covers 536 genes^3^. Following hybridization capture, libraries were sequenced deeply on a HiSeq 4000 (Illumina, San Diego, California) with 2 × 150 bp paired-end reads. Sequencing results were analyzed for single nucleotide variants using the CAPP-Seq bioinformatic pipeline^12,13^. CAPP-Seq was similarly performed on plasma with matched germline DNA^12–14^.

### Single nucleotide variant analysis from cfDNA

Only non-silent mutations with duplex support and with no germline support were considered when querying MRD from cfDNA^3^. Specifically, we defined maximum VAF as the maximum variant allele fraction among all non-silent mutations with duplex support detected by CAPP-Seq using our 145 kb driver gene-focused MRD gene panel^3^, regardless of the number of other mutations detected and their frequencies. Maximum VAF was selected as the metric representing tumor DNA by CAPP-Seq, and was correlated with MRD status in the surgical specimen. Non-silent SNVs in urine cfDNA with >2.3% VAF^3^ are represented in the Supplementary Fig. 3 heatmap. We additionally inferred tumor mutational burden using our urine CAPP-Seq results. Briefly, we utilized our TMB gene panel, which is 387 kb in size and covers 536 genes, and applied the equation determined previously by linear regression while accounting for potential dropout in order to infer exome-wide TMB^3^.

### Ultra-low-pass whole genome sequencing (ULP-WGS)

ULP-WGS libraries were prepared from 32 to 50 ng of sheared urine cfDNA using the Kapa HyperPrep kit (Roche, Basel, Switzerland). Libraries were balanced, pooled, and sequenced on a HiSeq 4000 (Illumina, San Diego, California) to a median deduplicated depth of 2x (Supplementary Data 6). FASTQ files were demultiplexed and raw reads were quality-filtered using fastp v.0.20.0. Quality-filtered reads were then aligned to the hg19 human genome assembly using BWA v.0.7.17. Aligned reads were deduplicated with Samtools v.1.13. ichorCNA v0.2.015^15^ was then used to infer tumor fractions in each urine cfDNA sample. Briefly, reads were summed in nonoverlapping bins of 10^6^ bases; local read depth was corrected for GC bias and known regions of low mappability, and artifacts were removed by comparison to ichorCNA’s built-in healthy control reference. Copy number alterations (CNAs) were then predicted across the whole genome using low tumor fraction parameters for cfDNA samples; X and Y chromosomes were excluded from copy number calculations. ichorCNA then used these binned, bias-corrected copy number values to model a two-component mixture of tumor-derived and non-tumor-derived fragments, from which it inferred the fraction of reads in each sample originating from the tumor (tumor fraction)^15^.

The visualization of aggregate genome-wide CNAs (Supplementary Fig. 2) was generated from compiled log_2_ ratios of copy number, broken down into three categories: No pathologic complete response (*n* = 39), pathologic complete response (*n* = 35), and healthy adults (*n* = 15). Following the removal of artifacts, regions were classified as exhibiting copy number gain if log_2_ of the copy number ratio was >0.58 (log_2_ (3/2)) or loss if log_2_ of the copy number ratio was <−1.0 (log_2_ (1/2))^16^. Midpoints of genes previously shown to be commonly altered in whole exome sequencing data of muscle-invasive bladder cancer, based on their annotation in Fig. 1 of the respective TCGA publications^4,5^ are specifically highlighted (Supplementary Figs. 2, 3).

### Machine learning model to predict pathologic complete response and survival

We implemented a random forest model for the prediction of pCR, which we validated using LOOCV. We used the maximum VAF, iTMB, and ULP-WGS-inferred tumor fraction (TFx) in urine cfDNA, which were combined together into one urine tumor DNA feature for the random forest model via multiplication followed by the square root of the product. Other features in the model included age, gender, ethnicity, smoking status, receipt of neoadjuvant chemotherapy, and tumor invasion status (Supplementary Fig. 5). We additionally developed another LOOCV random forest model using only urine cfDNA features (VAF, iTMB, and TFx) without the clinical variables (Supplementary Fig. 6). We used the Python scikit-learn package (v0.24.2)^17^ to implement the random forest algorithm, with the following parameters: n_estimators = 2000; criterion = gini; bootstrap = True. The performance of the model after LOOCV for predicting pCR was assessed by receiver operating characteristic (ROC) area under the curve (AUC) analysis.

Patients predicted by the LOOCV model to not achieve pCR were defined as MRD-positive, while those predicted to have pCR were defined as MRD-negative. LOOCV model MRD predictions were compared to gold-standard surgical pathology results (Fig. 1f) and were also stratified by Kaplan–Meier analysis from the time of surgical resection for progression-free survival (PFS) and overall survival (OS) (Fig. 2). The model was additionally generated using independent training and held-out validation cohorts (Supplementary Fig. 9). Furthermore, we calculated feature importance levels by assessing mean decrease in impurity^18^, to determine how classifications of pCR (MRD-negative) versus no pCR (MRD-positive) were affected if a particular feature was left out of the random forest model (Supplementary Fig. 5b).

### Power and statistical analyses

We powered the current study assuming a substantial difference in urine tumor DNA levels between patients who achieved pCR or healthy donors, compared to patients with no pCR. Assuming a large effect size estimated by Cohen’s *f* = 0.5, we accrued subjects to this study until there were at least 14 subjects per group (groups = healthy donors, bladder cancer with pCR, bladder cancer with no pCR) in order to detect a difference between healthy or pCR, and no pCR with an estimated power of 80% and significance level of 0.05 as determined by one-way ANOVA. Patient characteristics such as age, gender, ethnicity, smoking history, tumor stage, neoadjuvant chemotherapy, and histology were statistically compared between groups of pCR and no pCR patients using Fisher’s exact test for categorical variables and Student’s *t*-test for normally distributed continuous variables (Supplementary Data 7). SNV-derived maximum VAFs, inferred tumor mutational burden, and CNA-derived tumor fraction levels in urine cell-free DNA from patients with localized bladder cancer were statistically compared between groups of pCR and no pCR using the Mann–Whitney *U*-test (Fig. 1b–d and Supplementary Figs. 4a, 7a–c, 8a–c). The Python scikit-learn package (v0.24.2) was used for random forest modeling with LOOCV (Supplementary Figs. 5, 6) or with separate training and validation datasets (Supplementary Fig. 9). ROC analysis was carried out to assess the performance of the LOOCV random forest model and the corresponding AUC was calculated for the full cohort of 74 localized bladder cancer patients with and without pretreatment clinical variables (Fig. 1e and Supplementary Fig. 6b) and for MIBC patients (Supplementary Fig. 7d). MRD predictions based on the LOOCV random forest model were compared to surgical ground-truth by Fisher’s exact test (Fig. 1f and Supplementary Fig. 7e). Survival curves for PFS and OS were analyzed by the Kaplan–Meier method and statistical significance was determined by the log-rank test (Fig. 2 and Supplementary Figs. 7f-g, 8d-e, 9). The Mantel–Haenszel method was used to estimate hazard ratios. Cox proportional hazards model (PHM) univariate and multivariate analyses were developed to assess both PFS and OS (Supplementary Data 8, 9). In addition to random forest model prediction, hematocrit, body mass index, and urine cfDNA concentration were included in the multivariate models. For OS, there were no deaths during the follow-up period among patients predicted by the random forest model to achieve pCR. Given this, the assumption of proportional hazards was not met. We performed all Kaplan–Meier and Cox regression analyses starting from the time of surgery. The reverse Kaplan–Meier method was used to calculate the median follow-up time (Supplementary Data 1). All statistical analyses were performed using Prism 9 (GraphPad Software, San Diego, California) or SAS version 9.4 (SAS, Cary, North Carolina).

### Reporting summary

Further information on research design is available in the Nature Research Reporting Summary linked to this article.

## Supplementary information

Supplementary Figures

Supplementary Data

REPORTING SUMMARY

## Data Availability

All FASTQ files corresponding to sequenced patient samples are available from the sequencing read archive (SRA) under accession number PRJNA907063 and ID number 907063.
