# Supplementary material for: Urine cell-free DNA multi-omics to detect MRD and predict survival in bladder cancer patients
Source: NPJ Precis Oncol. 2023 Jan 19;7:6. doi: 10.1038/s41698-022-00345-w (PMC9852243; doi:10.1038/s41698-022-00345-w)
Supplement: Supplementary file 1 — Supplementary Figures [file 41698_2022_345_MOESM1_ESM.pdf]

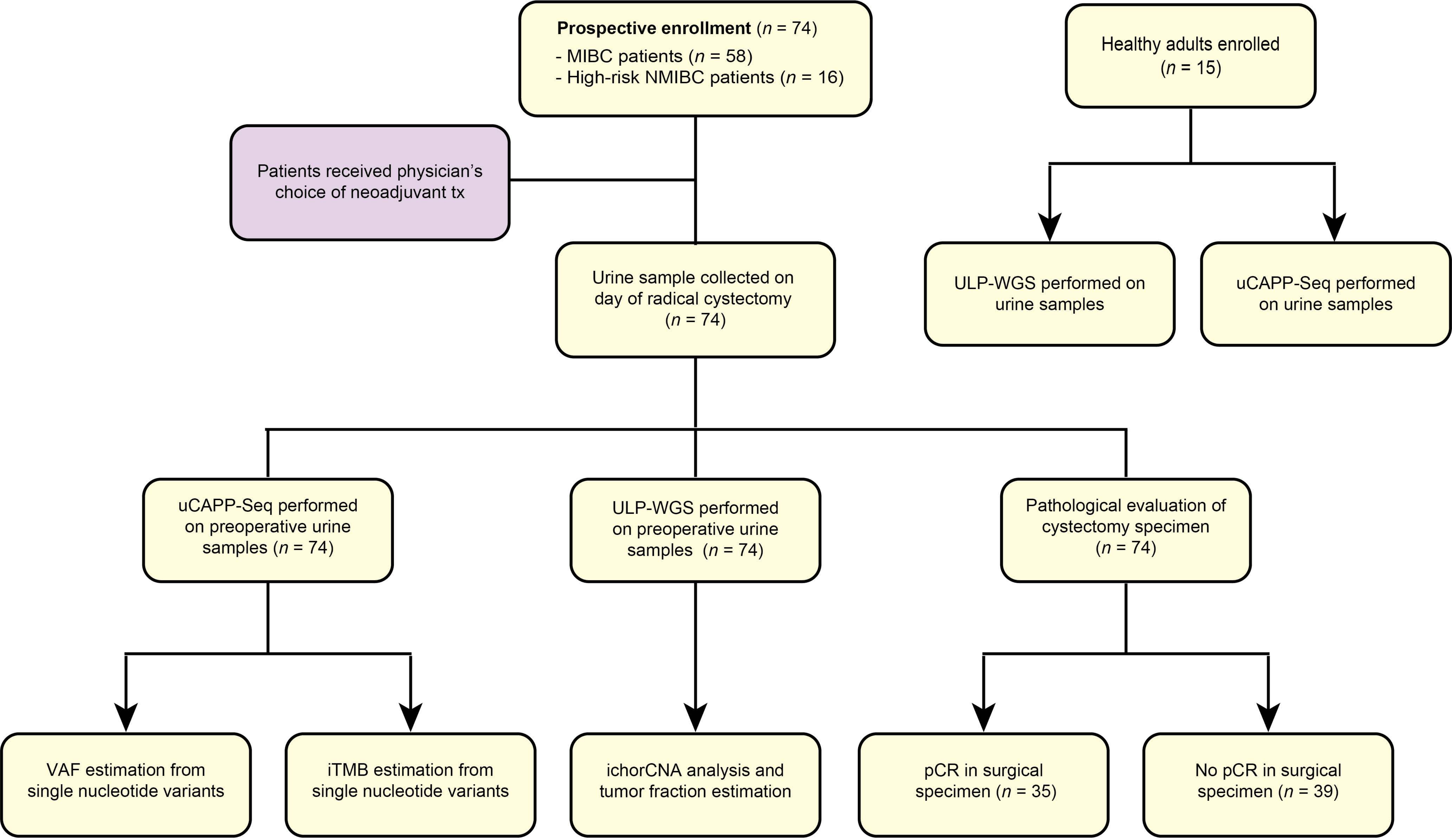

Supplementary Fig. 1 - Study schema. Patients with localized bladder cancer who were candidates for radical cystectomy were prospectively enrolled onto this study. Urine samples were then collected for uCAPP-Seq and ULP-WGS analysis as shown in the schema. Urine samples from 15 healthy adults were also used for ULP-WGS and uCAPP-Seq analysis. iTMB, inferred tumor mutational burden; MIBC, muscle-invasive bladder cancer; NMIBC, non-muscle-invasive bladder cancer; pCR, pathologic complete response; tx, treatment; uCAPP-Seq, urine Cancer Personalized Profiling by deep Sequencing; ULP-WGS, ultra-low-pass whole genome sequencing; VAF, variant allele frequency.

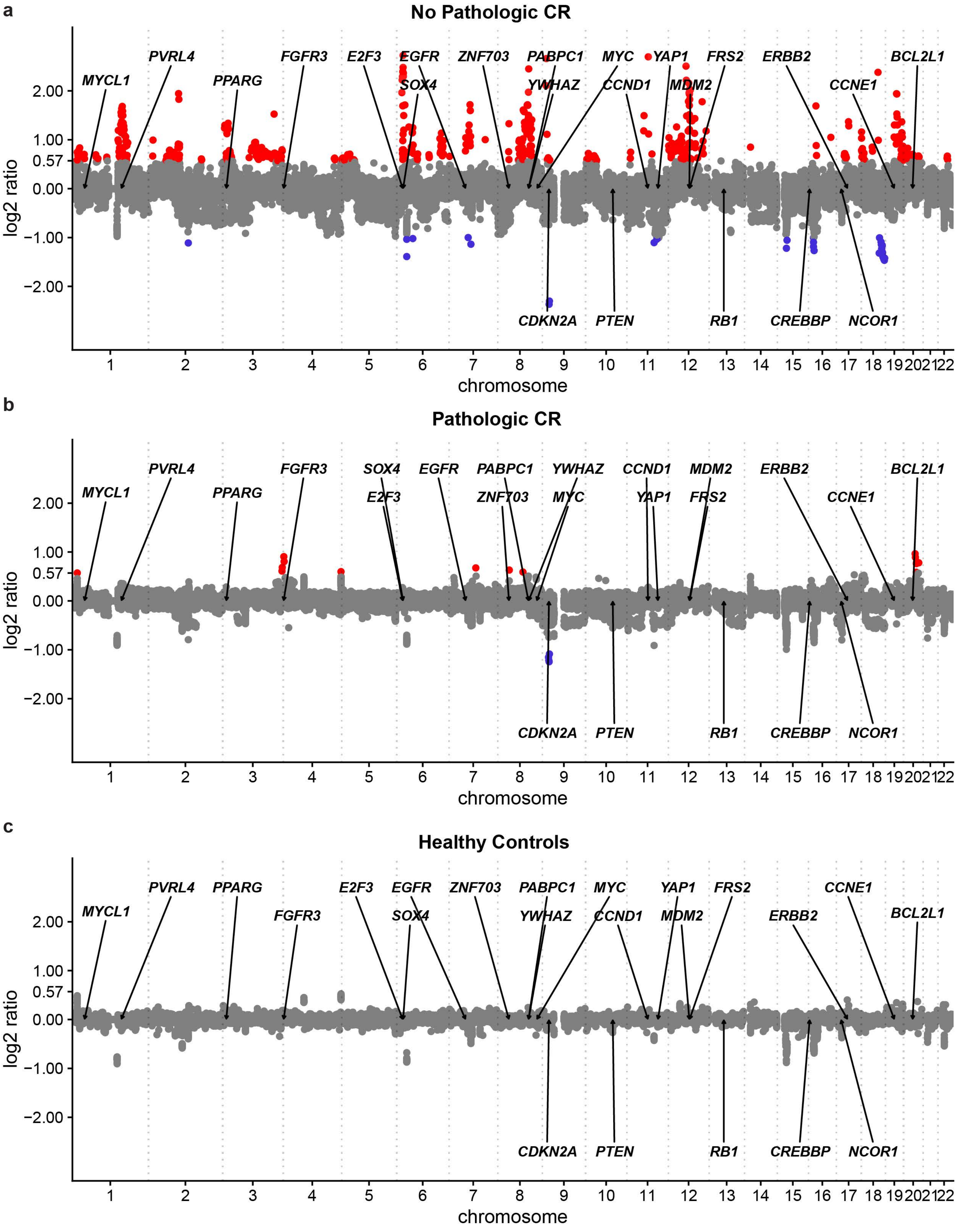

Supplementary Fig. 2 - Genome-wide copy number plots with annotation of genes important in bladder cancer. Plots represent the aggregate copy number alterations compiled from urine cell-free DNA data in (**a**) Patients with no pCR (n = 39), (**b**) Patients with pCR (n = 35) or (**c**) Healthy adults (n = 15). Each panel depicts log2 copy number ratios across the genome. Red represents copy number gain while blue represents copy number loss (Methods). Annotated genes are those previously reported in TCGA to be copy-number altered in bladder cancer (Methods). pCR, pathologic complete response; TCGA, the cancer genome atlas.

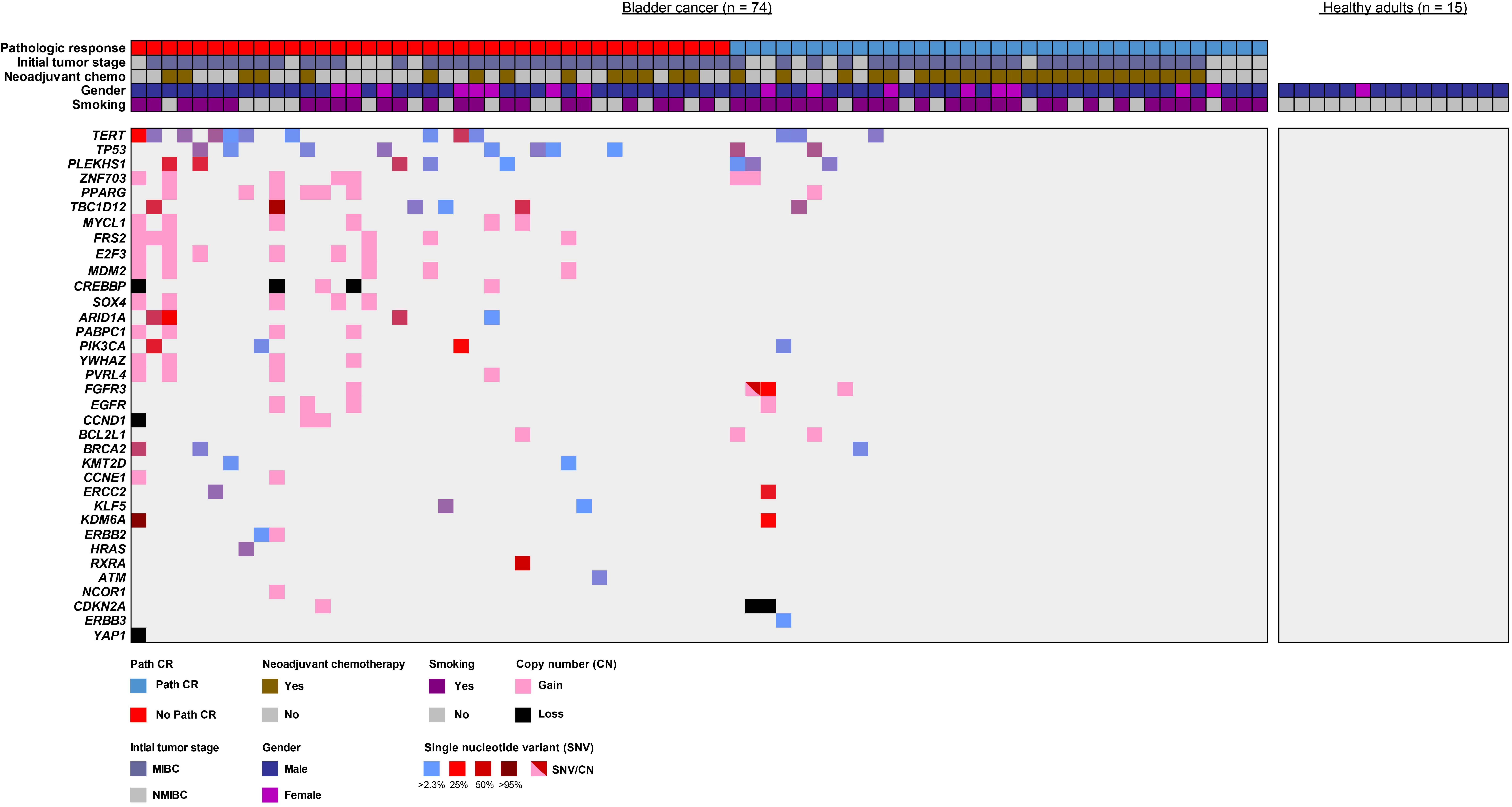

Supplementary Fig. 3 - Subject characteristics and detected genomic alterations. Co-mutation plot showing genomic alterations (mutations and copy number alterations) detected in pre-operative urine cell-free DNA from each patient with no pCR versus pCR and healthy adults. Mutational data represent non-silent SNVs detected within the MRD uCAPP-Seq gene panel, while copy number alterations represent ultra-low-pass whole genome sequencing data, focusing on genes reported by TCGA to be altered in muscle-invasive bladder cancer (Methods). Patient and healthy donor characteristics are represented by the upper heatmaps. NMIBC, non-muscle-invasive bladder cancer; MIBC, muscle-invasive bladder cancer; MRD, molecular residual disease; pCR, pathological complete response; SNV, single nucleotide variant; TCGA, the cancer genome atlas.

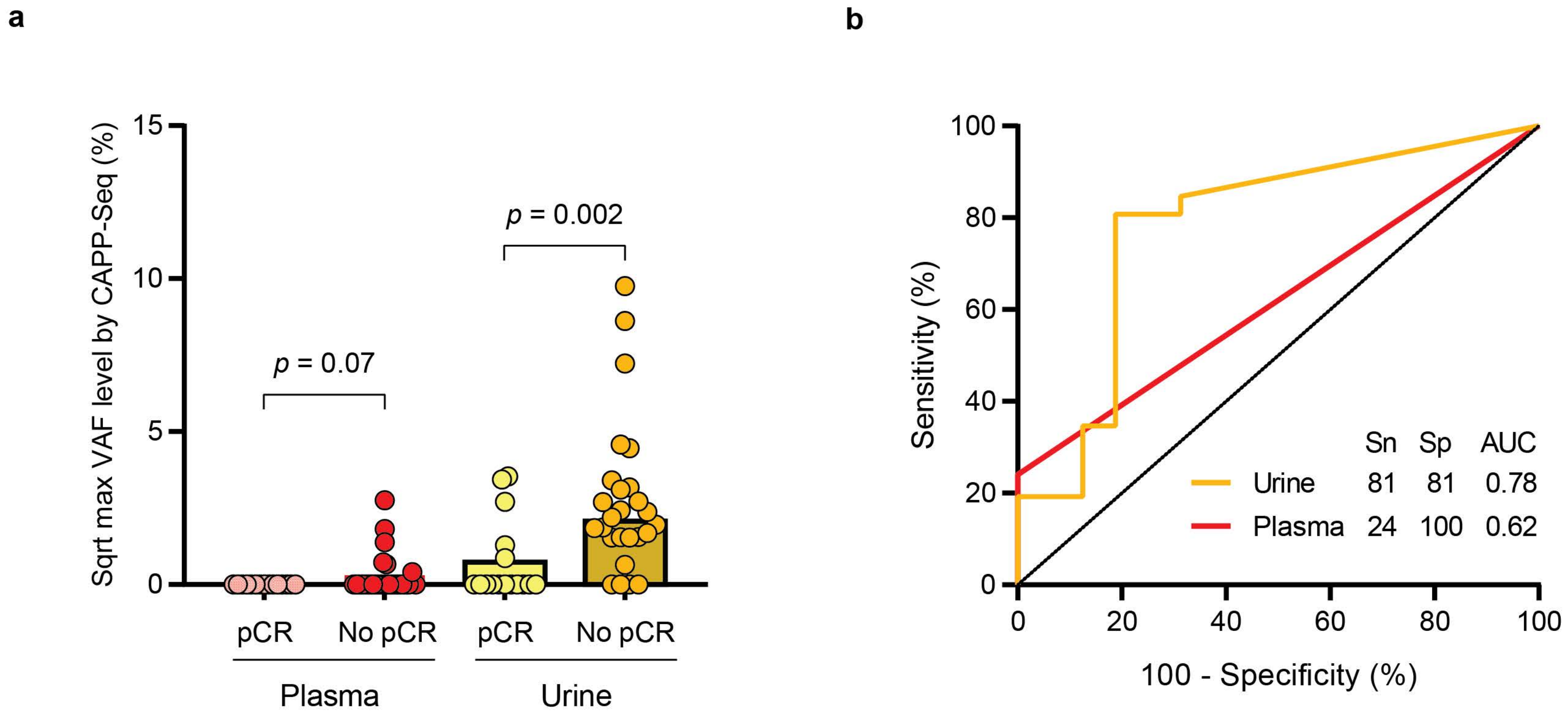

Supplementary Fig. 4 - Performance of CAPP-Seq in matched urine and plasma samples for detecting MRD and predicting pathologic response. (**a**) Scatter plot of maximum VAF levels after square-root transformation in urine versus plasma from 40 localized bladder cancer patients, compared to gold-standard surgical pathology. (**b**) ROC analysis for classifying pCR from no pCR patients by CAPP-Seq. CAPP-Seq in urine cell-free DNA classified pathologic response more accurately than in paired plasma (AUC 0.78 versus 0.62). AUC, area under the curve; CAPP-Seq, Cancer Personalized Profiling by deep Sequencing; MRD, molecular residual disease; pCR, pathologic complete response; ROC, receiver operating characteristic; Sqrt, square root; VAF, variant allele frequency.

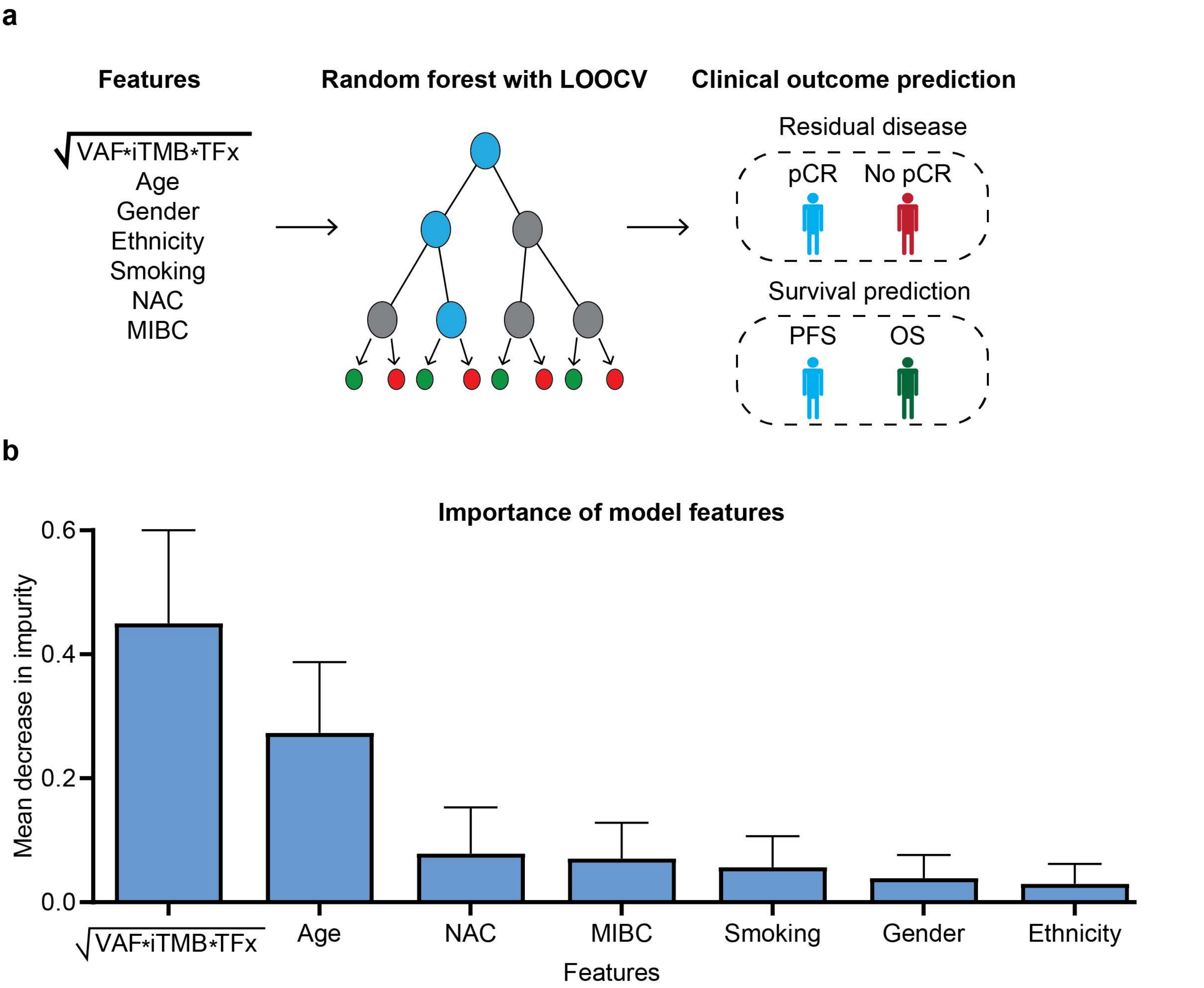

Supplementary Fig. 5 - Random forest model with LOOCV to predict pathologic complete response status. (**a**) Schema depicting the model's development, validation, and application. (**b**) Importance of features in the random forest model used for predicting pCR status. Error bars represent the standard deviation. iTMB, inferred tumor mutational burden; LOOCV, leave-one-out cross-validation; MIBC, muscle-invasive bladder cancer; NAC, neoadjuvant chemotherapy; OS, overall survival; pCR, pathologic complete response; PFS, progression-free survival; TFx, tumor fraction; VAF, variant allele frequency.

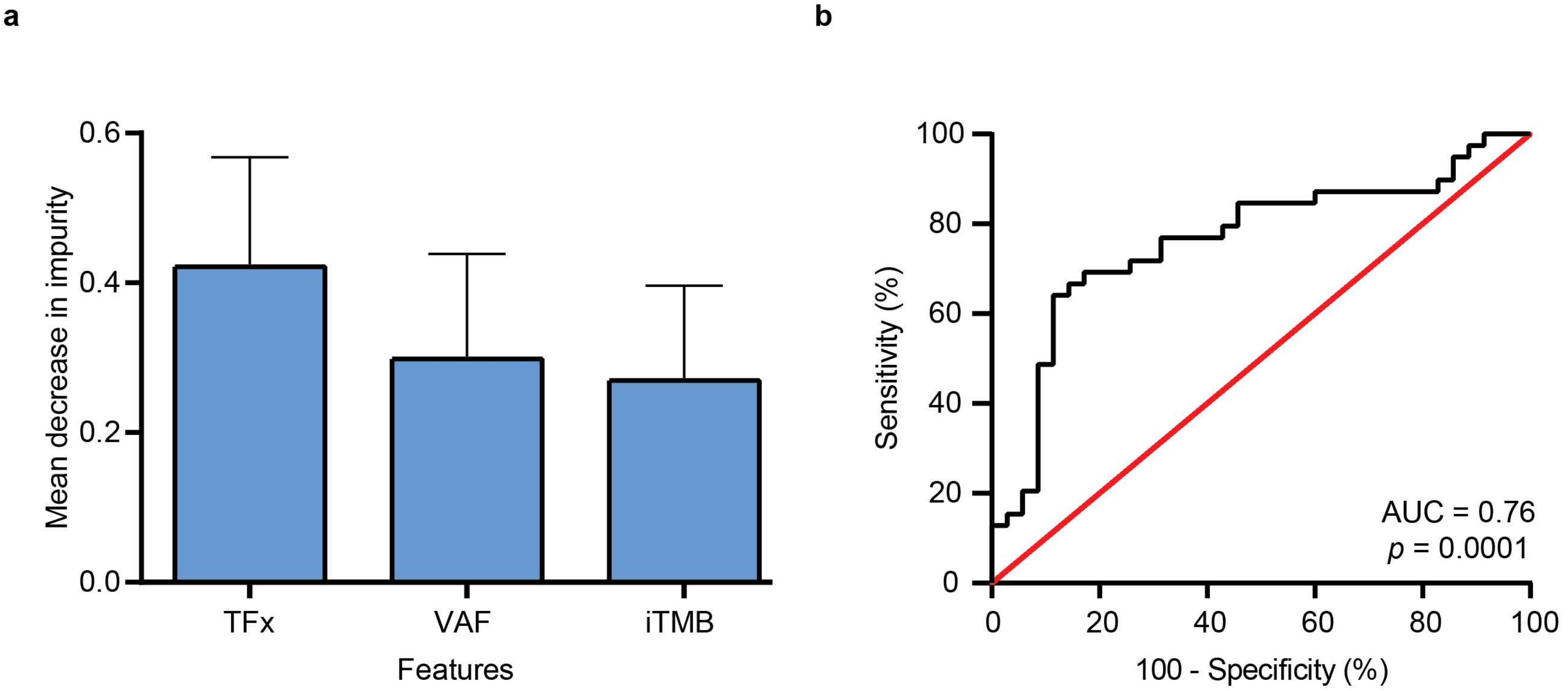

Supplementary Fig. 6 - Random forest model based on urine cell-free DNA features with LOOCV to predict pathologic complete response status. (**a**) Importance of features in the random forest model used for predicting pCR status based on urine cell-free DNA features only (TFx, maximum VAF and iTMB). Error bars represent the standard deviation. (**b**) ROC analysis of random forest model for predicting pCR after LOOCV (AUC = 0.76, p = 0.0001). AUC, area under the curve; iTMB, inferred tumor mutational burden; LOOCV, leave-one-out cross-validation; pCR, pathologic complete response; ROC, receiver operating characteristic; TFx, tumor fraction; VAF, variant allele frequency.

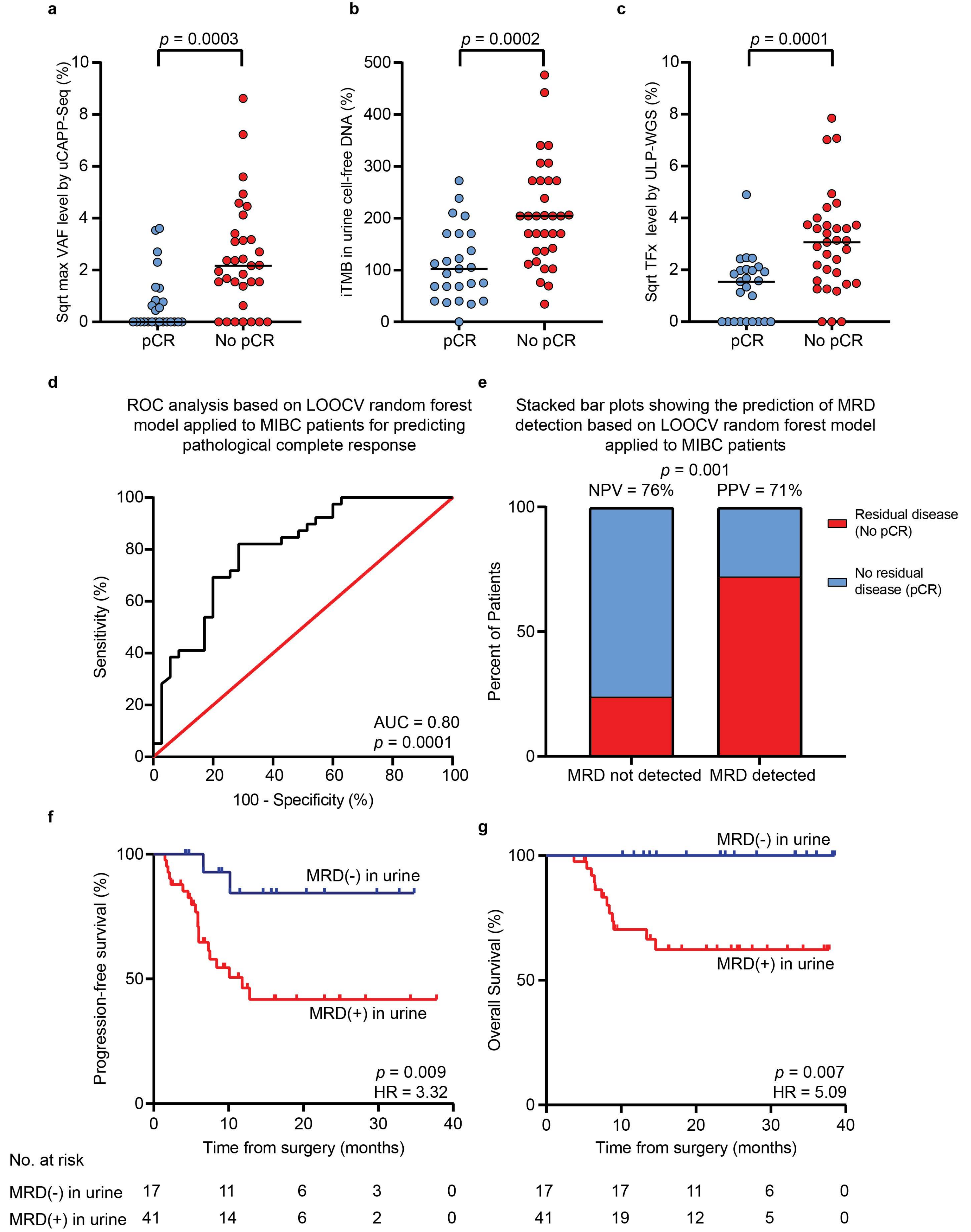

Supplementary Fig. 7 - LOOCV random forest model applied to MIBC patients to predict pathologic response and survival outcomes. Scatter plots displaying (**a**) maximum VAF (square-root transformed), (**b**) iTMB, and (**c**) TFx (square-root transformed), stratified by pathologic response status among MIBC patients (n = 58), with significance determined by the Mann-Whitney U test. (**d**) ROC analysis demonstrating the LOOCV random forest model's performance in classifying MIBC patients by pCR status; AUC of 0.80 (p = 0.0001). (**e**) Stacked bar plot depicting NPV and PPV of the LOOCV random forest model with significance determined by the Fischer's exact test. Kaplan-Meier analysis of (**f**) progression-free survival and (**g**) overall survival based on the LOOCV random forest model applied to patients with MIBC (n = 58). p values were calculated by the log-rank test and HRs by the Mantel-Haenszel method. AUC, area under the curve; cfDNA, cell-free DNA; iTMB, inferred tumor mutational burden; LOOCV, leave-one-out cross-validation; MIBC, muscle-invasive bladder cancer; MRD, molecular residual disease; NPV, negative predictive value; pCR, pathologic complete response; PPV, positive predictive value; ROC, receiver operating characteristic; Sqrt, square root; TFx, tumor fraction; VAF, variant allele frequency.

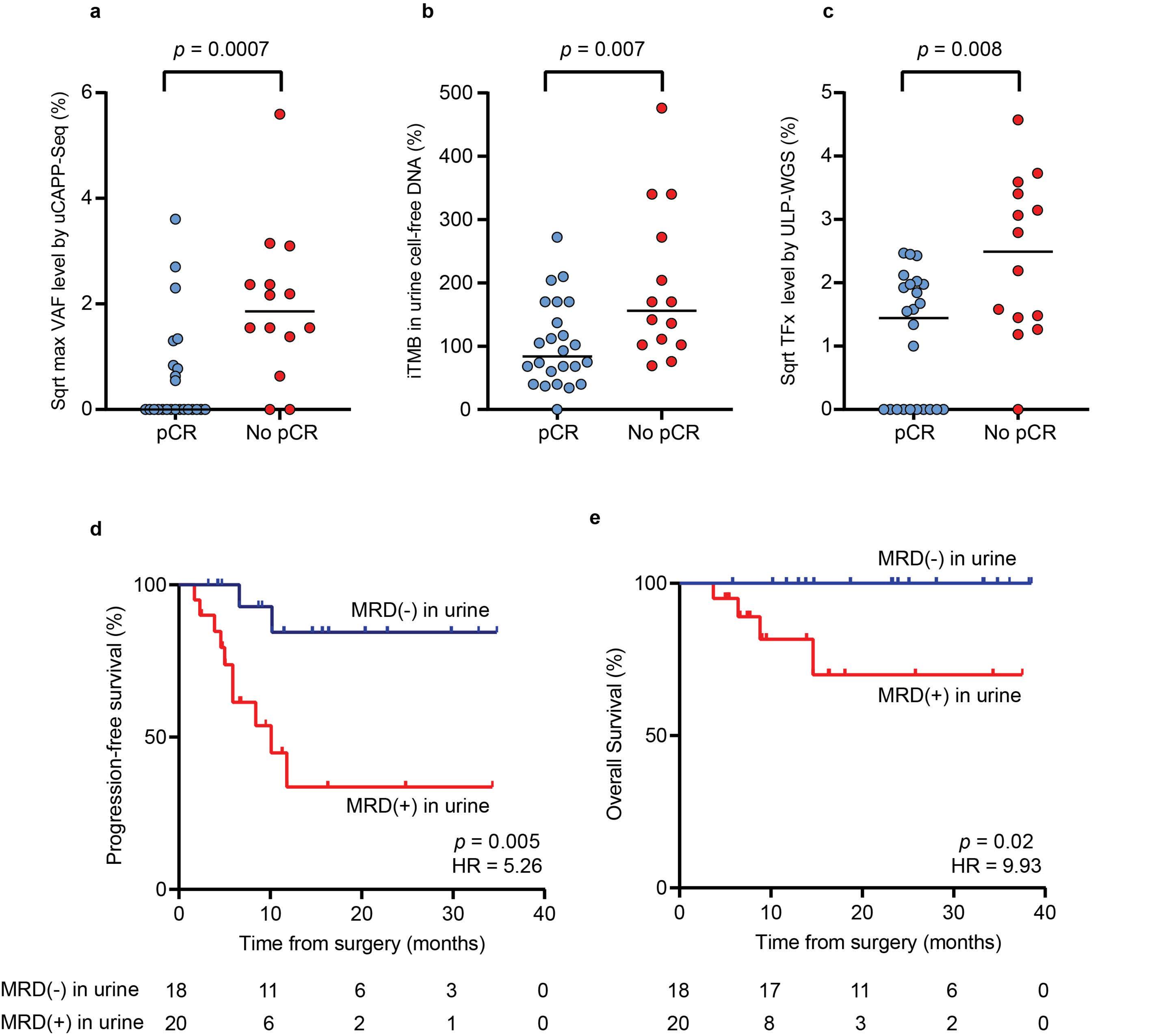

Supplementary Fig. 8 - LOOCV random forest model applied to MIBC patients who received neoadjuvant chemotherapy to predict pathologic response and survival outcomes. Scatter plots displaying (**a**) maximum VAF (square-root transformed), (**b**) iTMB, and (**c**) TFx (square-root transformed), stratified by pathologic response status among MIBC patients who received NAC (n = 38). Significance was determined by the Mann-Whitney U test. Kaplan-Meier analysis of (**d**) progression-free survival and (**e**) overall survival based on the LOOCV random forest model applied to MIBC patient who received NAC. *p* values were calculated by the log-rank test and HRs by the Mantel-Haenszel method. cfDNA, cell-free DNA; iTMB, inferred tumor mutational burden; MIBC, muscle-invasive bladder cancer; MRD, molecular residual disease; NAC, neoadjuvant chemotherapy; pCR, pathologic complete response; Sqrt, square root; TFx, tumor fraction; VAF, variant allele frequency.

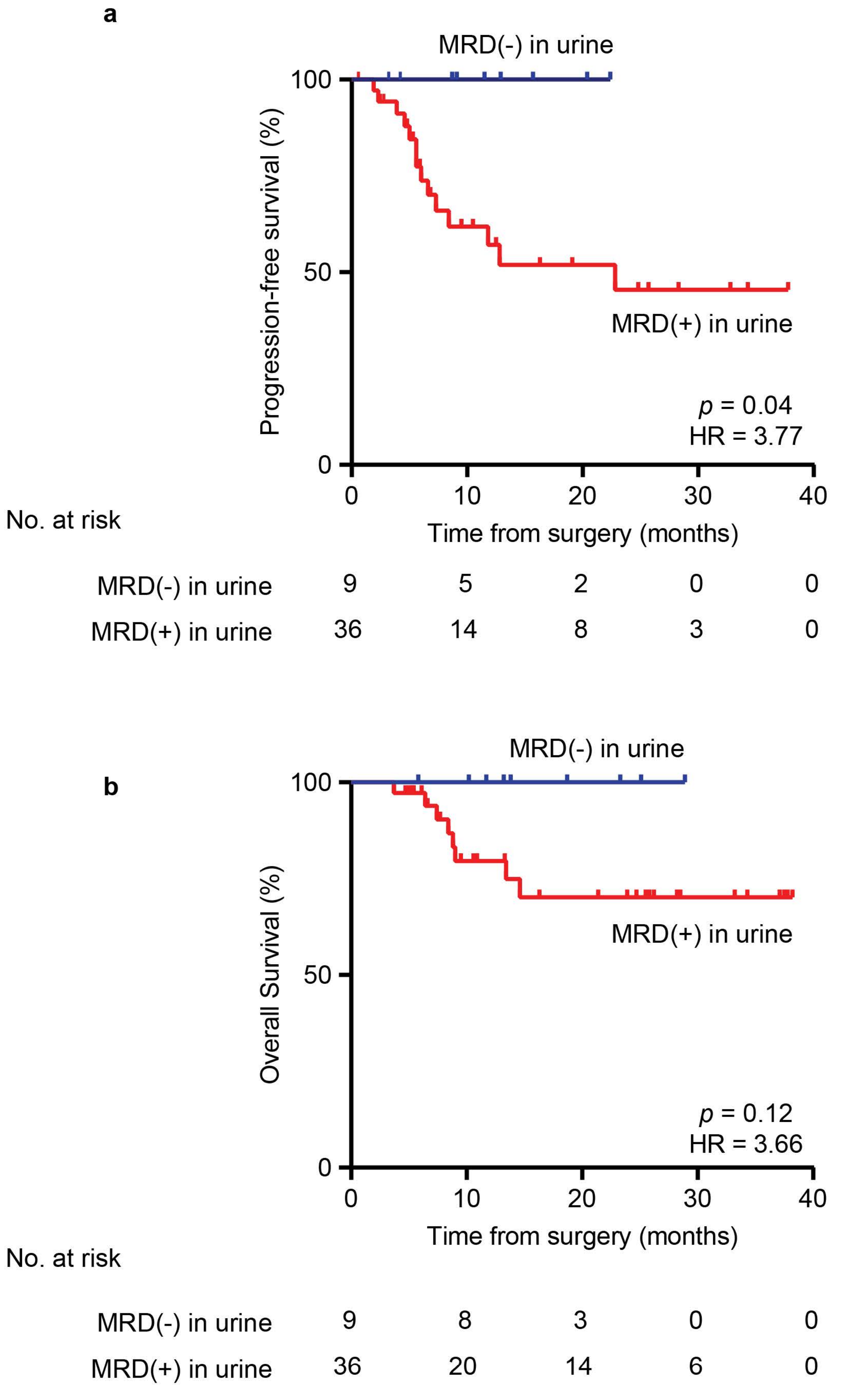

Supplementary Fig. 9 - Random forest model evaluated for survival outcomes in a held-out validation cohort. Kaplan-Meier analysis of (**a**) progression-free survival and (**b**) overall survival in a held-out validation cohort of 45 localized bladder cancer patients, after random forest model training using data from 29 localized bladder cancer patients (Methods). *p* values were calculated by the log-rank test and HRs by the Mantel-Haenszel method. HR, hazard ratio; MRD, molecular residual disease.
