# Supplementary material for: Urine cell-free DNA multi-omics to detect MRD and predict survival in bladder cancer patients
Source: NPJ Precis Oncol. 2023 Jan 19;7:6. doi: 10.1038/s41698-022-00345-w (PMC9852243; doi:10.1038/s41698-022-00345-w)
Supplement: Supplementary file 3 — REPORTING SUMMARY [file 41698_2022_345_MOESM3_ESM.pdf]

nature portfolio

Corresponding author(s): Aadel A. Chaudhuri

Last updated by author(s): Dec 1, 2022

Reporting Summary

Nature Portfolio wishes to improve the reproducibility of the work that we publish. This form provides structure for consistency and transparency in reporting. For further information on Nature Portfolio policies, see our Editorial Policies and the Editorial Policy Checklist.

Statistics

For all statistical analyses, confirm that the following items are present in the figure legend, table legend, main text, or Methods section.

n/a	Confirmed	
☐	☒	The exact sample size (*n*) for each experimental group/condition, given as a discrete number and unit of measurement
☐	☒	A statement on whether measurements were taken from distinct samples or whether the same sample was measured repeatedly
☐	☒	The statistical test(s) used AND whether they are one- or two-sided<br>*Only common tests should be described solely by name; describe more complex techniques in the Methods section.*
☐	☒	A description of all covariates tested
☐	☒	A description of any assumptions or corrections, such as tests of normality and adjustment for multiple comparisons
☐	☒	A full description of the statistical parameters including central tendency (e.g. means) or other basic estimates (e.g. regression coefficient) AND variation (e.g. standard deviation) or associated estimates of uncertainty (e.g. confidence intervals)
☐	☒	For null hypothesis testing, the test statistic (e.g. *F*, *t*, *r*) with confidence intervals, effect sizes, degrees of freedom and *P* value noted<br>*Give P values as exact values whenever suitable.*
☒	☐	For Bayesian analysis, information on the choice of priors and Markov chain Monte Carlo settings
☒	☐	For hierarchical and complex designs, identification of the appropriate level for tests and full reporting of outcomes
☐	☒	Estimates of effect sizes (e.g. Cohen's *d*, Pearson's *r*), indicating how they were calculated

Our web collection on statistics for biologists contains articles on many of the points above.

Software and code

Policy information about availability of computer code

Data collection	No specific software was used for data collection beyond software associated with commercially available hardware (Agilent 2100 Bioanalyzer v.B.02.11.S1824), Illumina HiSeq 4000 control software). Primary processing of sequencing data was performed with BWA (v.0.7.17-r1188), samtools (v.0.1.18 and v.1.13), and bedtools (v.2.30.0). For ULP-WGS data, sequencing reads were quality-filtered using fastp v.0.20.0. aligned using BWA v.0.7.17, and deduplicated with Samtools v.1.13. ichorCNA v.0.2.015 was used to calculate tumor fractions. For SNV calling, we utilized the CAPP-Seq with iDES pipeline (https://cappseq.stanford.edu/).
Data analysis	Software packages used for data analysis in this study are detailed in the Methods section including ichorCNA v.0.2.015, Python scikit-learn package (v.0.24.2), GraphPad Prism 9 (v.9.3.1), and SAS v.9.4.

For manuscripts utilizing custom algorithms or software that are central to the research but not yet described in published literature, software must be made available to editors and reviewers. We strongly encourage code deposition in a community repository (e.g. GitHub). See the Nature Portfolio guidelines for submitting code & software for further information.

Data

Policy information about availability of data

All manuscripts must include a data availability statement. This statement should provide the following information, where applicable:

- Accession codes, unique identifiers, or web links for publicly available datasets
- A description of any restrictions on data availability
- For clinical datasets or third party data, please ensure that the statement adheres to our policy

All de-identified data related to this manuscript are available from the corresponding author upon reasonable request.

Human research participants

Policy information about studies involving human research participants and Sex and Gender in Research.

Reporting on sex and gender	Sex is included as a clinical variable in the random forest model for the prediction of molecular residual disease. Among 74 patients in the study cohort, 58 (78%) were male and 16 (22%) were female.
Population characteristics	Patients were required to be at least 18 years old and to have a diagnosis of bladder cancer confirmed by histologic or cytologic assessment.
Recruitment	Patients and healthy donors were enrolled onto NCT04354064 (ClinicalTrials.gov).
Ethics oversight	Ethical oversight was provided by the Institutional Review Board within the Human Research Protection Office at the Washington University in St. Louis.

Note that full information on the approval of the study protocol must also be provided in the manuscript.

Field-specific reporting

Please select the one below that is the best fit for your research. If you are not sure, read the appropriate sections before making your selection.

☒ Life sciences ☐ Behavioural & social sciences ☐ Ecological, evolutionary & environmental sciences

For a reference copy of the document with all sections, see nature.com/documents/nr-reporting-summary-flat.pdf

Life sciences study design

All studies must disclose on these points even when the disclosure is negative.

Sample size	We powered the current study assuming a substantial difference in urine tumor DNA levels between patients with pCR compared to those without pCR. Based on a large effect size estimated by Cohen's f = 0.5, we accrued subjects onto the study until there were at least 14 subjects per group (i.e., healthy donors, pCR, and no pCR) to detect a difference with 80% power, as determined by a one-way ANOVA with a significance level of 0.05.
Data exclusions	All analyzed sequencing data generated in this work satisfied quality control thresholds.
Replication	All attempts at replication were successful.
Randomization	As this was an observational study, no randomization of subjects was performed.
Blinding	Analysis of radical cystectomy specimens for residual disease was performed using AJCC 8 criteria as part of the standard clinical workflow, blinded to the urine tumor DNA-based predictions.

Reporting for specific materials, systems and methods

We require information from authors about some types of materials, experimental systems and methods used in many studies. Here, indicate whether each material, system or method listed is relevant to your study. If you are not sure if a list item applies to your research, read the appropriate section before selecting a response.

Materials & experimental systems

n/a	Involved in the study
☒	☐ Antibodies
☒	☐ Eukaryotic cell lines
☒	☐ Palaeontology and archaeology
☒	☐ Animals and other organisms
☐	☒ Clinical data
☒	☐ Dual use research of concern

Methods

n/a	Involved in the study
☒	☐ ChIP-seq
☒	☐ Flow cytometry
☒	☐ MRI-based neuroimaging

Clinical data

Policy information about clinical studies

All manuscripts should comply with the ICMJE guidelines for publication of clinical research and a completed CONSORT checklist must be included with all submissions.

Clinical trial registration	NCT04354064 (ClinicalTrials.gov)
Study protocol	Institutional Review Board protocol #201411135 and #201903142
Data collection	Patients were enrolled between 2019 and 2021.
Outcomes	The primary outcome of the study was pathologic complete response (pCR) versus no pCR, as assessed by surgical pathology of the radical cystectomy specimen. Secondary outcomes were progression-free survival and overall survival.
